# Maxillofacial gunshot injures and their therapeutic challenges: Case series

**DOI:** 10.1002/ccr3.2827

**Published:** 2020-04-13

**Authors:** Mehrnoush Momeni Roochi, Farnoosh Razmara

**Affiliations:** ^1^ Department of Maxillofacial Surgery Tehran University of Medical Sciences Tehran Iran

**Keywords:** bone grafting, craniomaxillofacial gunshot injury, reconstructive surgery

## Abstract

Maxillofacial gunshot injuries require proficiency to determine a suitable treatment plan and surgical intervention. In this paper, present 4 gunshot cases. Treatment in these patients is very challenging. Moreover, post‐treatment infections are a serious problem in such cases. Thus, step‐by‐step surgery is essential to obtain a better result in these patients.

## Graphical abstract

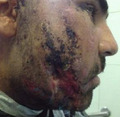

## 1 INTRODUCTION

Facial trauma results from several causes, such as motor vehicle/cycle accidents, assaults, suicides and of course gunshots.^1^, ^2^

Gunshot wounds cause extensive damages to both soft and hard tissues, representing a complex set of challenges for a maxillofacial surgeon, thus requiring their skill, patience, long‐term follow‐up, and eventually their numerous surgical procedures.^3^, ^4^

The severity of the injuries in gunshot wounds depends on many factors such as the caliber of the weapon; the distance from which the patient has been shot; and the size, shape, and velocity of the shrapnel as well as the jagged edge of the fragment. Accordingly, the associated wounds are classified into penetrative (a wound accompanied by disruption of the body surface that extends into the underlying tissue or into bony cavity), avulsive (a wound that happens when skin is torn from your body during an accident or other injury), and perforative (an injury in which an object enters the body or a structure and passes all the way through), the most complicated of which is certainly the avulsive type due to tissue loss.^3^, ^5^, ^6^ Definitive treatment of gunshot wounds has still remained controversial; they can be treated immediately, early, and delay. Some authors have referred to the treatment of gunshot wounds in the upper and midface with early reconstruction but submental wounds in delayed, although no definitive results have been reported.^7^ As we know, an appropriate management consists of three stages: 1. initial stabilization, 2. definitive reconstruction, and 3. secondary refinement.^8^, ^9^

The point is that for each case in this category, we have an individual approach, guideline, and order of treatment. Perhaps, we need to generalize and standardize our treatment plan. With this in mind, in this article we try to present four cases injured by gunshot wounds, bring forward the treatment modalities for each case, and discuss their associated problems.

## 2 CASES PRESENTATION

This case series was conducted on four cases of gunshot wounds referred to Sina hospital, Tehran, Iran. All patients underwent clinical and paraclinical assessments. Three‐dimensional CT‐scan was taken from all patients, and primary medical evaluations were performed.

### 2.1 Case 1

**Figure 1:**
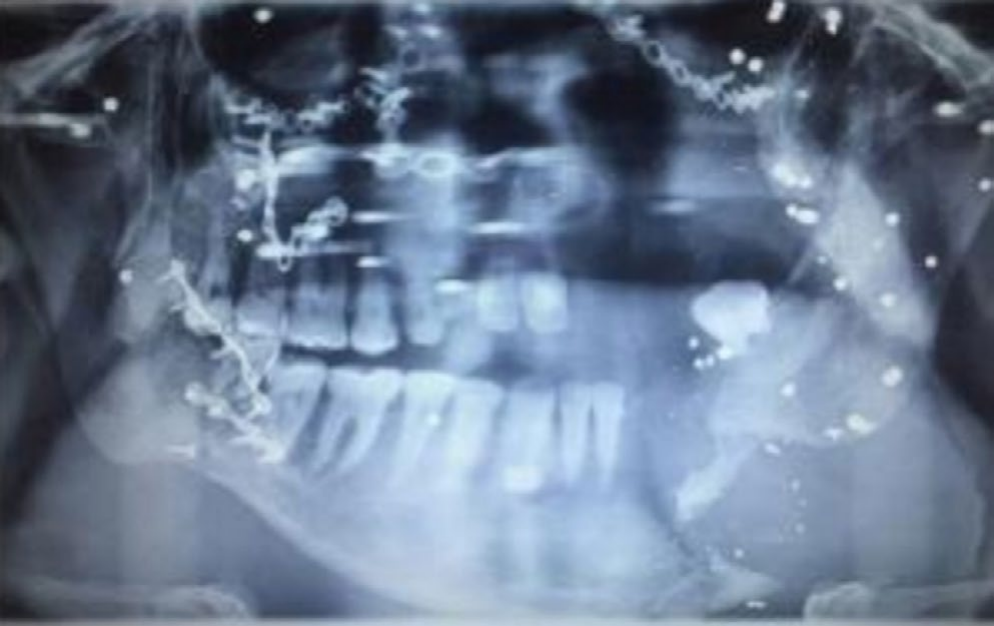
Mandibular fracture

**Figure 2:**
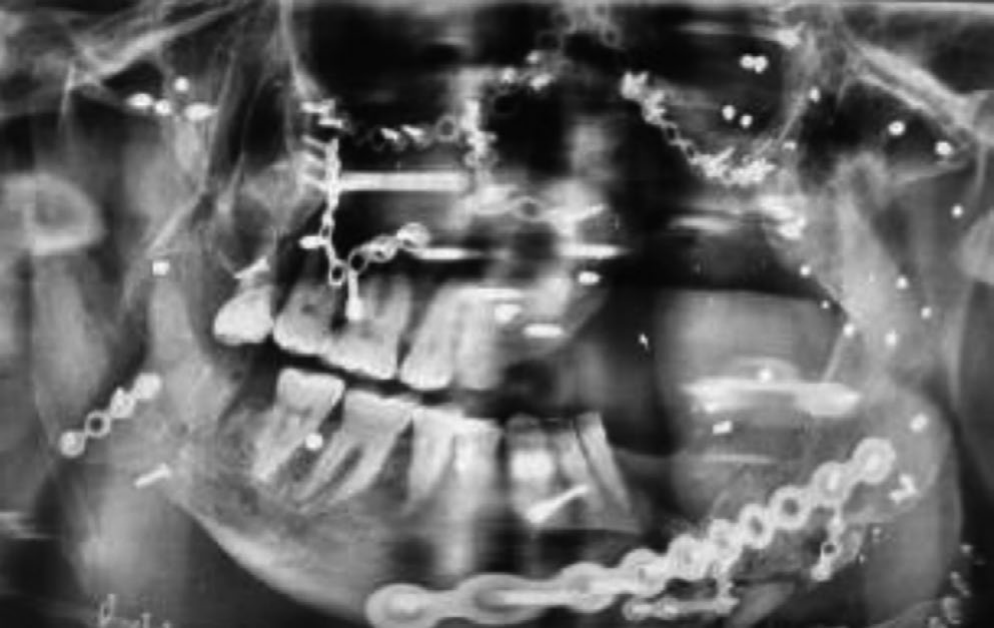
Reduction of mandibular

A 34‐year‐old man with a history of suicide attempt by a gun was referred to the hospital one month after trauma. The patient suffered from midface and mandibular fractures and deformity of his face (Figure 1). His injury was penetrative form. Midface and Right of the mandible reconstruction were done through his first surgery by the another surgeon one month after trauma. Six months later, during his second surgery, mandibular reconstruction in the left side was performed. Submandibular (Apron) approach was used to access the fracture segments on the left side, after osteotomy we reduced fracture segments and fixed by a reconstruction plate and two miniplates. On the right side, two miniplates that fixed in the first surgery had become loose, were removed, and replaced with a four‐hole mandibular plate (Figure 2). As third stage one year later, reconstructive surgery has been done for the patient's nose.

### 2.2 Case 2

**Figure 3:**
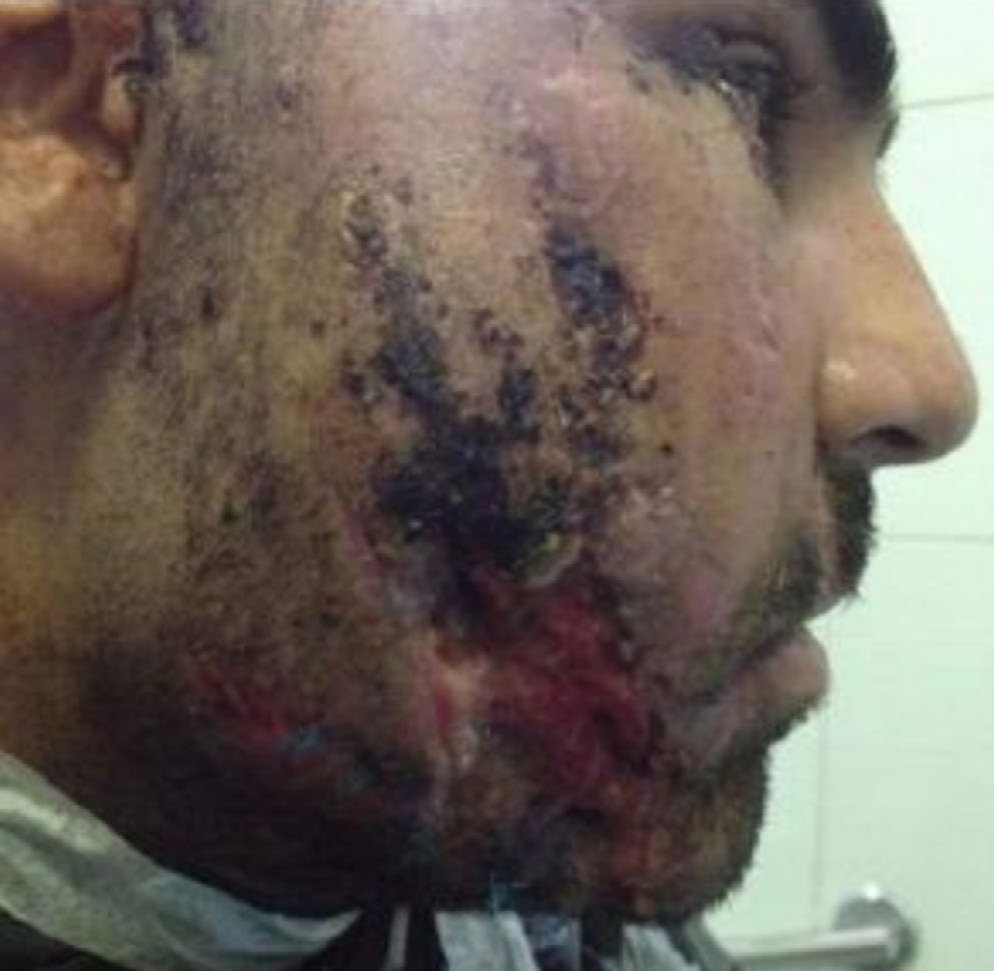
Avulsive injuries of soft tissue and skin mandible

A 32‐year‐old man with a diagnosis of right comminuted mandibular fracture, right comminuted zygomaticomaxillary complex fracture, right mandibular dentoalveolar fracture, and avulsive injuries of soft tissue and skin at his mandible due to gunshot after 18 days was hospitalized, and his injury was in category of penetrative and avulsive wound (Figure 3). Right eye had become no light perception (NLP). Through his first surgery, it was decided to treat the fracture of right zygomaticomaxillary complex. Because of avulsive injuries at the mandible, we could not treat the mandible at the same time. After general anesthesia, hemicoronal incision done on the right side, to access the lateral orbital wall, body of zygoma and zygomatic arch. Via a subciliary incision, we had access to infra. Orbital rim and orbital floor and the segments have been reduced and fixed with two four‐hole miniplates. Moreover, Medpor prosthesis was placed on the eye floor to reconstruct the defect. To the zygomatic buttress which has been reduced and through vestibular approach we had an access, and fractures fixed with two miniplates, arch bar and IMF has been used for temporary treatment of mandible fracture. Canfield's dressing with honey was used for avulsive soft tissue to regeneration.

**Figure 4:**
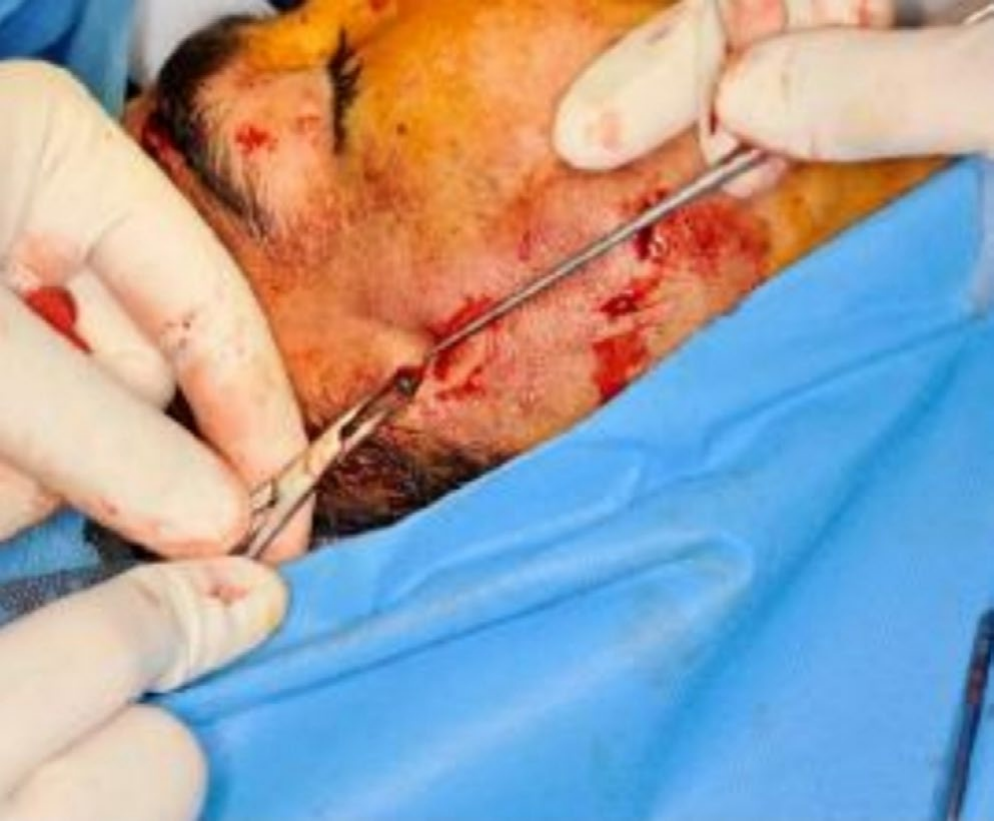
Bullets were extracted from the maxillofacial area

**Figure 5:**
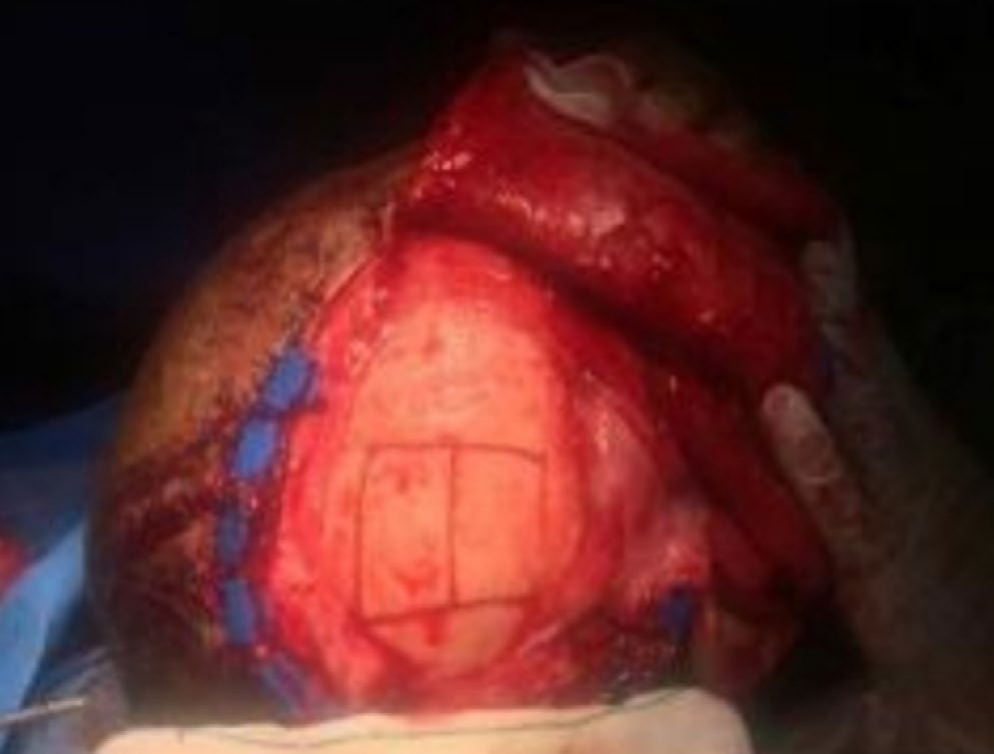
Preparation of calvarium

**Figure 6:**
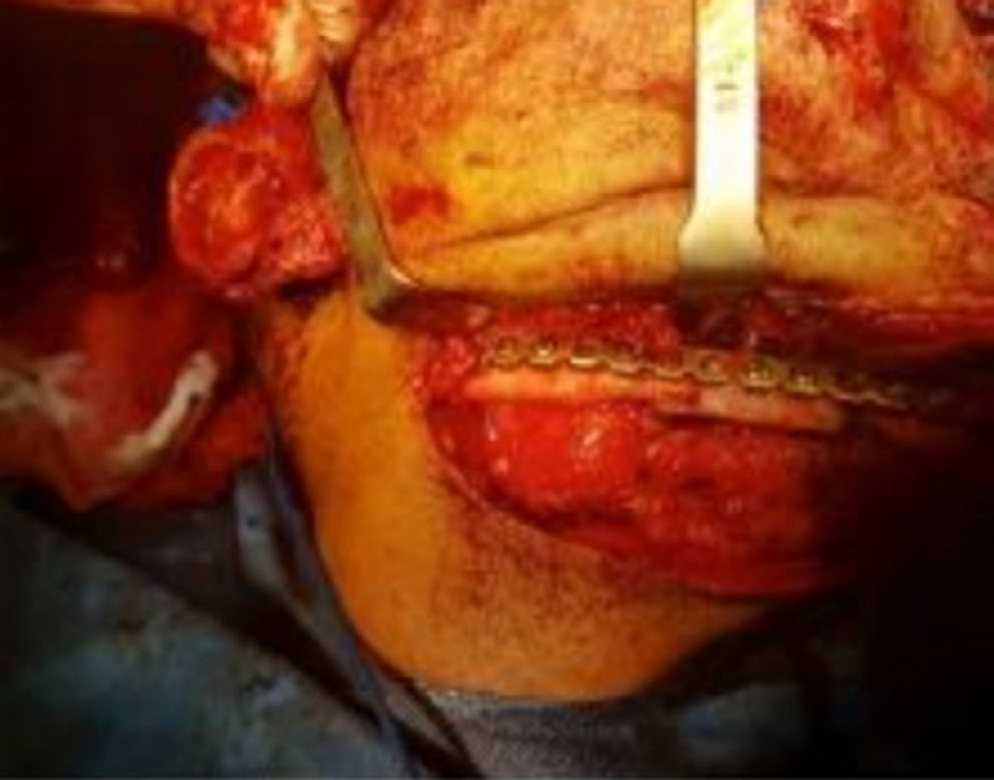
Bone defect was reconstructed by calvarial bone grafts

Eight months after the first surgery, the second surgery was performed to remove the bullets from the maxillofacial area and reconstruct the mandible. A total sum of nine bullets were extracted from the maxillofacial area with the aid of navigation (Figure 4). Then, the arch bars in two jaws were applied; after osteotomy of fracture segments, the teeth were manipulated in appropriate occlusion, and the fragments were fixed with a reconstruction plate and four miniplates. One fragment was fixed with a lag screw. Hence, the mandibular was fixed properly. Thereafter, the patient had the problem of bulky soft tissue in malar and buccal areas due to fibrosis after repair of avulsed soft tissue. One year after the second surgery, all plates in midface were removed to reduce the prominence of the cheek, the bone was shaved at zygomatic area, and debulking of the soft tissue of the cheek was carried out. Furthermore, there was a nonunion in mandible due to bone resorption bone defect causing the nonunion was reconstructed by calvaria bone grafts (Figures 5, 6) Since the patient had facial nerve paresis and sagging of the lip at the corner of the mouth, the muscles of the corners of the lips were lifted upward and fixed.

### 2.3 Case 3

**Figure 7:**
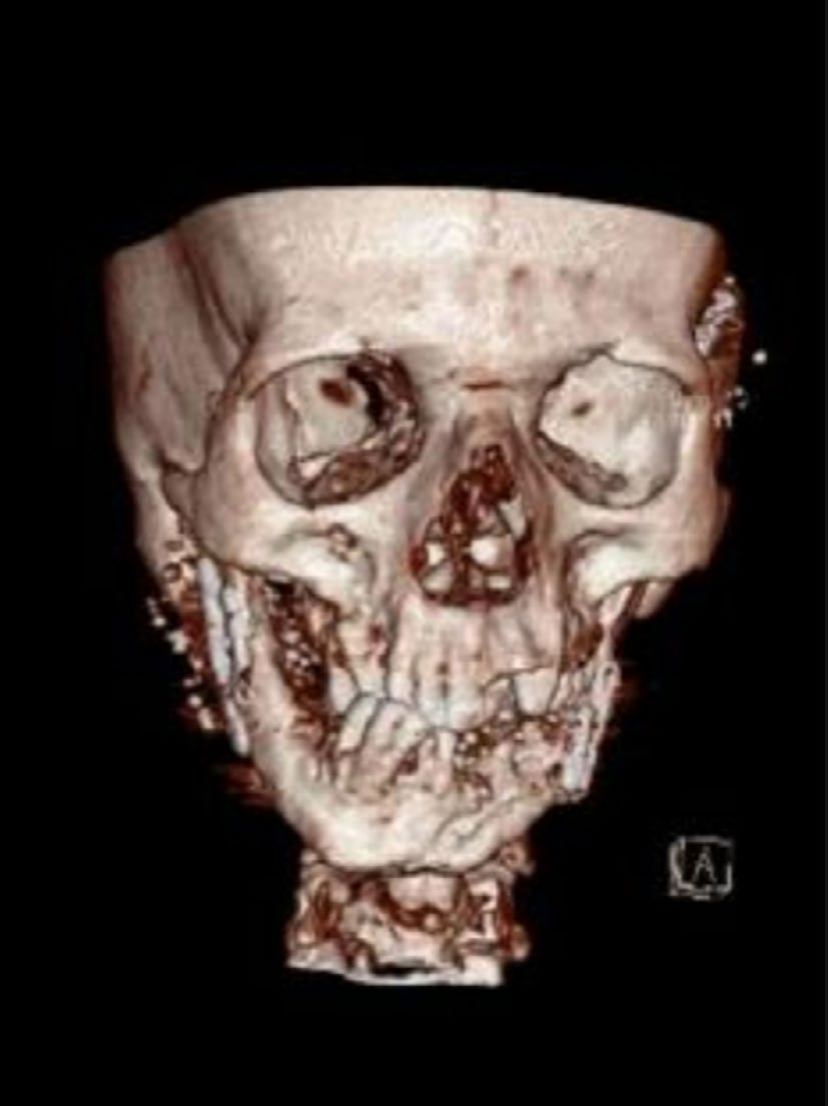
Malposition of mandibular fracture segments

**Figure 8:**
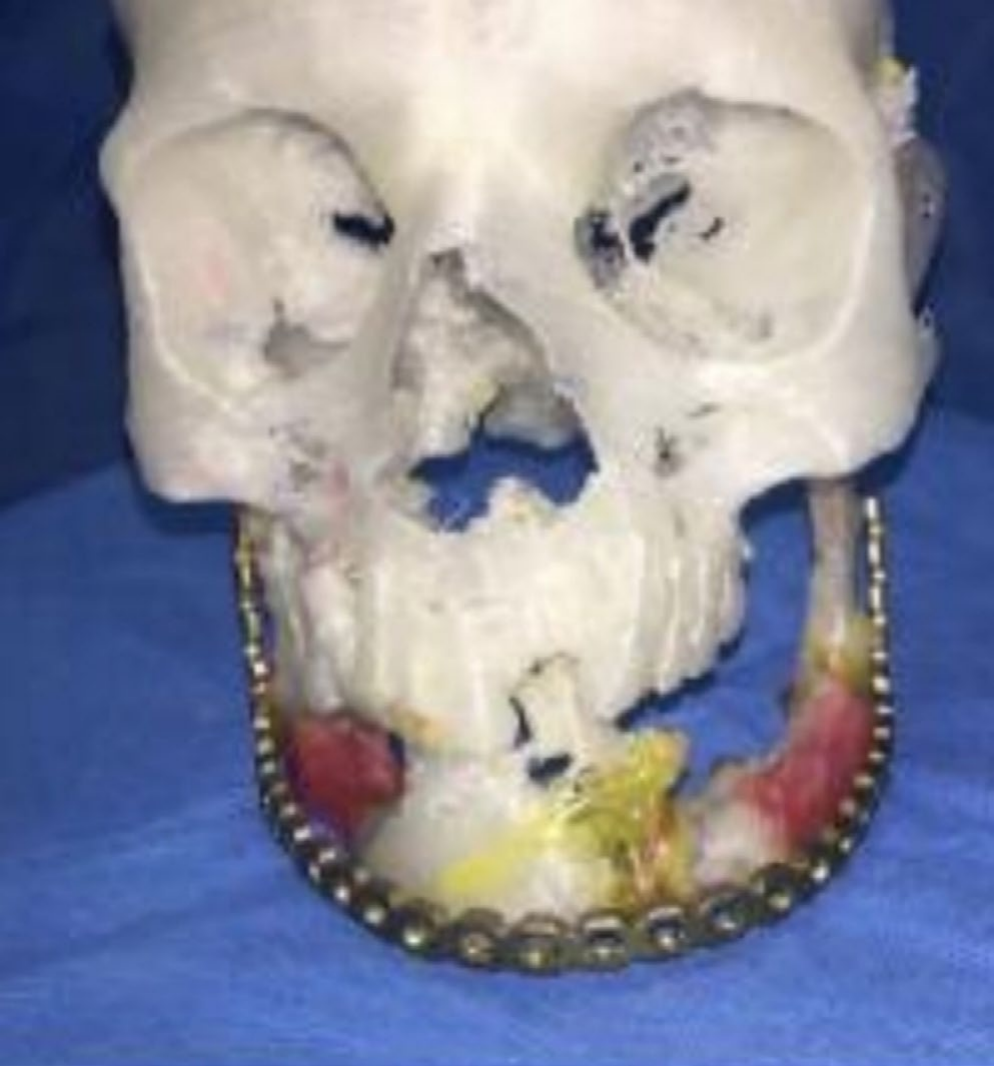
The misplaced mandibular fragments were fixed in an appropriate position on the stereolithographic model

**Figure 9:**
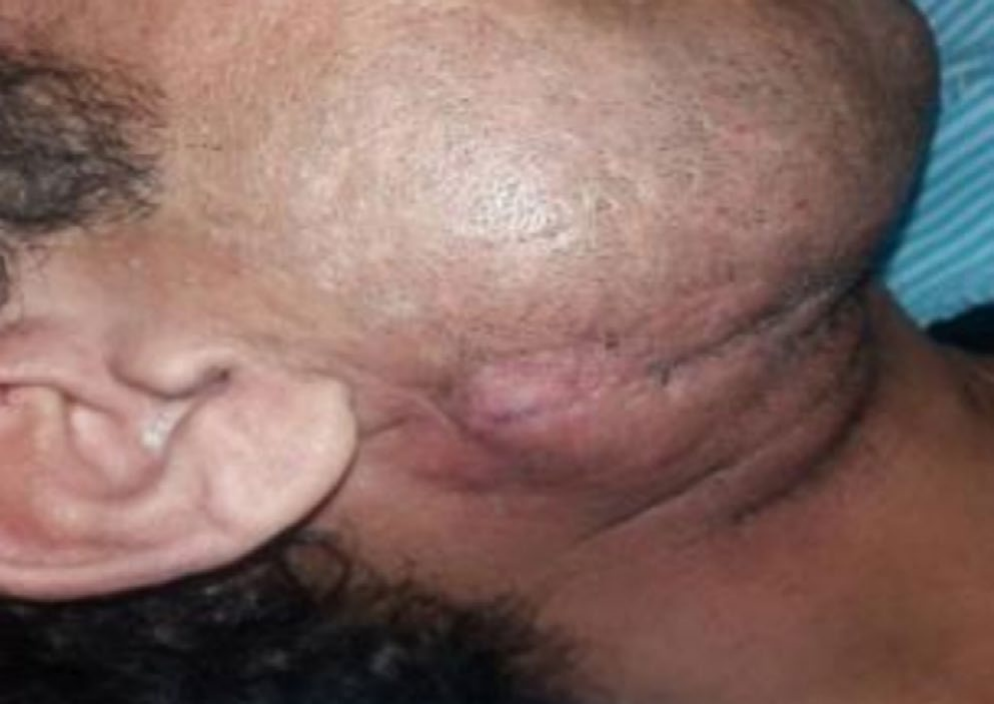
Infection in the place of surgery

A 31‐year‐old male patient with penetrative wound, who had been shot two years ago, had an inappropriate fixation surgery in a another center six months ago, and his chief compliant was mandibular deviation to the right, mandibular deficiency in the left side, class III occlusion, and malposition of mandibular fracture segments (Figure 7). Paraclinical evaluation has been done whole skull and face; stereolithographic model was prepared from the patient's face and skull. The misplaced mandibular fragments were fixed in an appropriate position on the model, and the reconstruction plate was placed on the reconstructed model (Figure 8). The patient underwent general anesthesia, and mandible was accessed through extraoral apron incision, the previous plates were removed, and osteotomy of the malpositioned segments has been done. Moreover, bilateral coronoidectomy was done. The displaced ramus segments were positioned properly, reconstruction plate was fixed in a specified point, and bone defects were reconstructed by iliac graft and coronoid. Finally, class I occlusion was obtained for the patient. The incision was sutured in three layers. Unfortunately, the patient suffered from a postoperation infection after 14 days (Figure 9). Culture and sensitivity test (C&S) was carried out for the patient, and it was determined that the infection was caused by a gram‐negative *E coli*. Tissue debridement as well as removement of the loose screw was done. Antibiotic therapy was by intervenous Tazosin 4.5 gr was prescribed in order to control the infection.

### 2.4 Case 4

**Figure 10:**
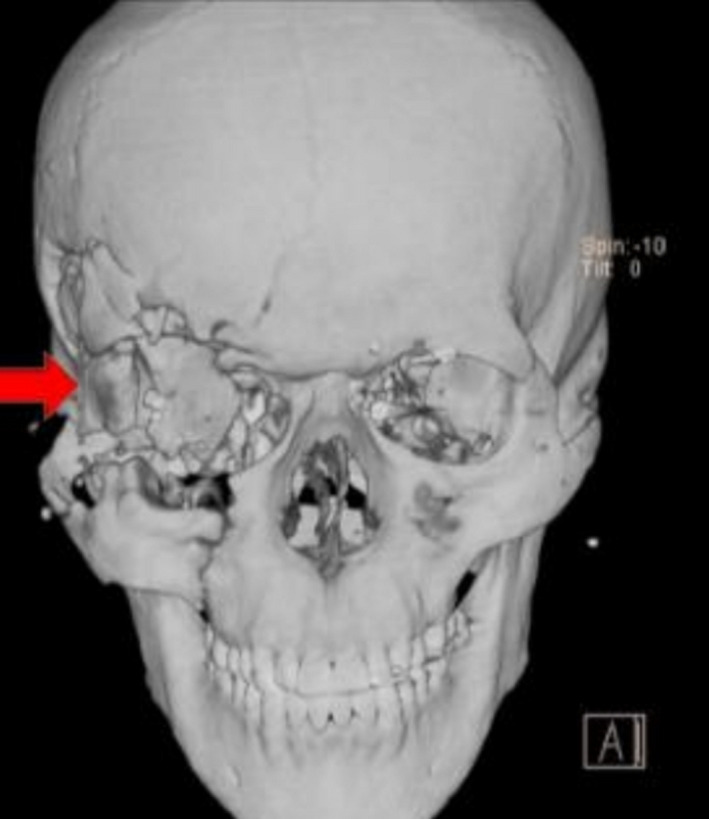
Fractures in the upper right side of the face

**Figure 11:**
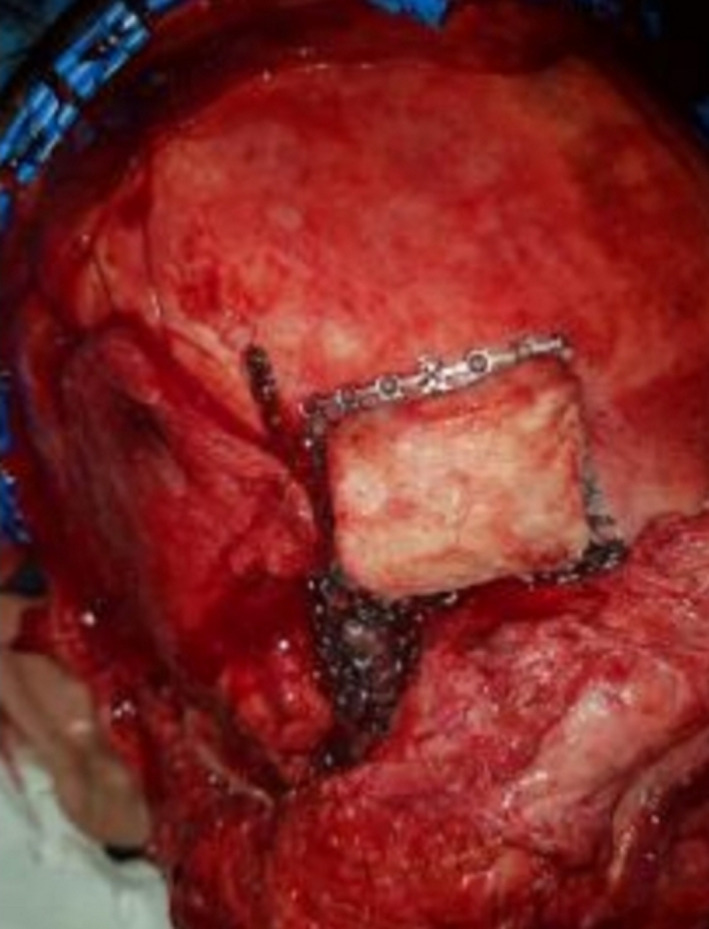
Titanium mesh was totally covered by gala and pericranium layer

**Figure 12:**
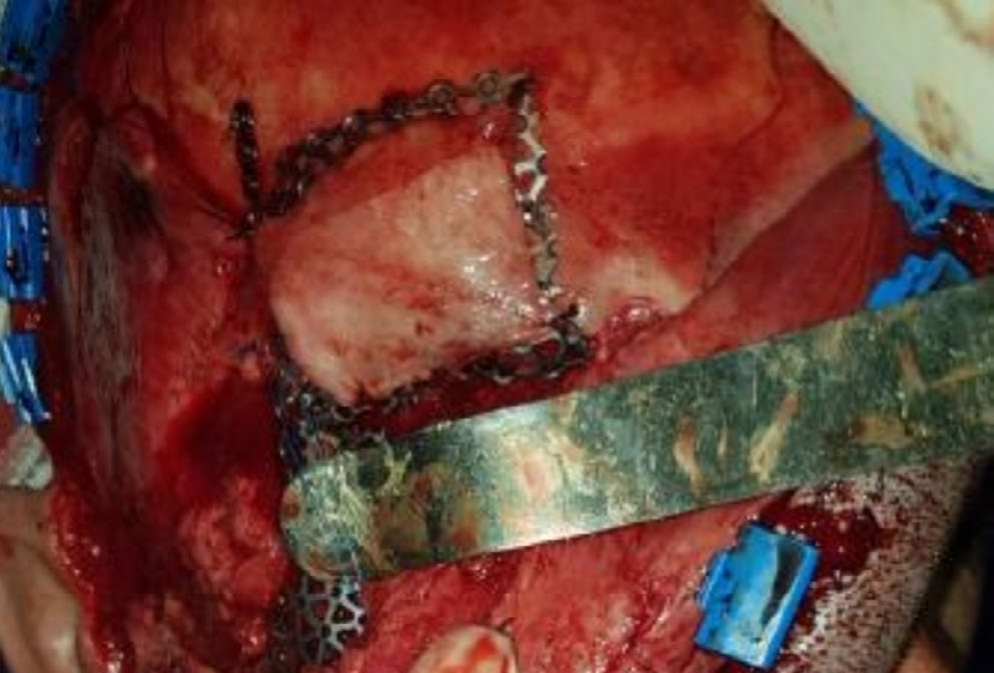
Suspension suture

A 27‐year‐old man injured by gunshot 4 months ago was referred suffering from zygomaticomaxillary complex, zygomatic arch, superior orbital rim fracture on his right side, and frontal and temporal bone fracture on the same side. His wound was penetrative type (Figure 10). Bicoronal, transconjunctival with lateral canthotomy and maxillary vestibular approaches were used to make access to these fractures. Fortunately, the dura was remained intact, and all of the mentioned fractures were fixed by miniplate. Orbital floor was reconstructed by titanium mesh, and the titanium mesh applied in superior orbital rim was totally covered by gala and pericranium layer (Figure 11). Since soft tissue suspension is necessary in gunshot patients, suspension suture in order of lift the malar soft tissue was done in this case as well (Figure 12).

## 3 DISCUSSION

The actual prevalence of facial gunshot is unknown, but it has been reported that 6% of gunshot wounds locate in the maxillofacial region.^10^ In addition, 50% of suicide attempts, 14% of assaults, and 12% of accident injuries have been reported to take place in this area and 22% of maxillofacial gunshot wound (MGSWs) include mandibular gunshots.^11^

To detect fractures in an area, using conventional radiography is adequate for the initial evaluation. However, locating the exact position of the missile in these two‐dimensional radiographs is controversial.^12^ Recently, the three‐dimensional types, computed tomography (CT), and CBCT have been greatly beneficial. CT is the gold standard and represents the proximity of the projectile path and the injury related to critical structures. In fact, it presents one‐millimeter axial views from the cranium to the bottom of the mandible.^6^, ^9^, ^12^

Another option is ultrasonography which is accurate, safe, and cost‐effective. Furthermore, it displays the position of the projectile which presents important tissue damages during the surgery.^13^, ^14^ By using Stereolithographic model and three‐dimension prints, the defects could be analyzed easier. Through using these systems, it is possible to create mirror imaging, and the patient's defect can be modeled according to the intact side. By doing so, we have the best treatment plan and result for reconstruction. In this study, this method was applied in one of the cases which led to a desirable result.

Surgical management consists of three stages:

Debridement
Fracture stabilization
Primary closure.^5^, ^15^, ^16^

At the first stage, management of the involved soft tissue includes decontamination and debridement of the wound as well as removal of all loose fragments, projectiles, and nonviable tissues.^1^, ^5^, ^6^, ^17^, ^18^ since removal of all projectiles is neither necessary nor good practice as it means extensive surgical wound. Projectiles that are easily accessible should be removed. Also those that are within the tongue and near joints or vessels should be removed because of the risk of contamination and vessel wall erosion from movement.^19^ Khatib et al describe sequence computer‐aided craniomaxillofacial reconstruction in 6 stages: (1) midface/orbital reconstruction, (2) oromandibular reconstruction, (3) palatomaxillary reconstruction, (4) internal orbital reconstruction, (5) soft tissue reconstruction—lip, nose, etc, and (6) dental rehabilitation.^20^

As for extensive and contaminated wounds, the best choices are irrigation with pulsed lavage system and prophylactic antibiotic therapy (immediately before surgery and continuing through the procedure, but not more than 24 hours postprocedure).^1^, ^6^, ^9^ Antibiotic choices can be penicillin, cephalosporin and clindamycin that prescribed according to the circumstances.^21^ After debridement, the critical plan involves skeletal fixation and reconstruction of comminuted bone. The main objective at this time is to restore the anteroposterior projection and the width of the face. Plating the zygomatic arch is the other guide.^6^, ^7^ In cases with fractured mandible, it is initially important to regain mandibular continuity and occlusion.^6^, ^7^, ^22^ Fixation of fractured bone can be done by external and/or internal plates. Internal plates can be used in fractures with larger bone fragments which can embrace the screws. Miniplates are used in the cranium and midface, but mandible typically accepts the large locking plates (2.4 mm).^6^, ^23^ According to AO/ASIF principles, in cases without extensive facial fractures and infections, plate osteosynthesis can be used along with debridement and primary closure.^24^ Bone grafts can be used in defects larger than 5 mm in the midface and mandible regions.^25^, ^26^ Iliac crest bone is typically used in mandibular defects. The appropriate options for midface defects are iliac crest, cranium, and rib.^6^, ^27^ Iliac bone grafts are properly used in defects without soft tissue requirement. They minimize donor‐site disease and prevent resorption. Another option for mandibular defects is the use of synthetic bone grafts.^28^

According to our experience, the health of soft tissue in the recipient site is much more important than the location where the bone graft is harvested.

In gunshot patients, it is better to apply a microvascular flap because in these defects, soft tissue often does not have a good quality. Free fibular osteocutaneous flap is a standard method to reconstruct mandibular defects larger than 6 cm.^29^

To obtain an optimal esthetic and a functional result, soft tissue reconstruction is so important in order to prevent infection. However, the best result comes from concurrent bony‐soft tissue reconstruction.^30^ One of the important complications after gunshot wound treatment is nonunion of bone segments.^31^ The lack of proper tissue healing and nonunion or malunion is common in these patients. If delayed healing is more than 8 weeks, may be nonunion occurred. Reasons of nonunion are multifactorial: osteomyelitis, edentulous mandible, alcohol and drug abuse, delayed treatment, teeth in the fracture line, improper reduction, and poor fixation are among the causes. Nonunion's sign almost pain, abnormal mobility in the fractured segment and malocclusion after treatment.^32^ Radiographs demonstrate large bone gap, no evidence of healing and, in later stages, show rounding off of the bone ends. Also, it has been suggested that even without maxillomandibular fixation, patients must be encouraged to regain motion, hygiene, and nutrition.^33^ Sometimes nonunion cases may be converted to delayed union caused by immobilization. However, open reduction is recommended when conservative treatment fails. The recommended protocol for the operative treatment of nonunion in the mandible is as follows: an extraoral approach, debridement of the infected and necrotic tissues down to the healthy and bleeding bone, placement of a rigid reconstruction plate, and use of bone substitute materials when necessary.^34^

An important point in gunshot patients is soft tissue hyperplasia or hypoplasia in these areas for which we can use debulking or injecting fat or filler in the defected area. In one of the cases, we noticed soft tissue hyperplasia, and by debulking, the tissue improved the patient's appearance. Enophtalmos, lid retraction, and trismus are the most common delayed problems in patients with MGSWs treated by standard methods.^9^, ^35^

Additionally, another important issue which causes lots of challenges in such patients is the infection which occurs after the treatment.^36^ It can be caused by bony sequestration, hopeless teeth, loose screws in the area, and opportunistic infections (as a result of patient's being hospitalization for long time). Even choosing suitable antibiotics to control their infections is challenging. We experienced such difficulties in the cases presented.

According to the experience, step‐by‐step treatment of these patients offers the best results. However, due to mental problems of these patients, since most of them have attempted suicide before visiting the doctor, they are truly difficult to manage. In earnest, it should be noted to postinfection of treatment, most probably because of lack of soft tissue. We will try to manage this feature in future.

## CONFLICT OF INTEREST

None declared.

## AUTHOR CONTRIBUTIONS

MMR: Patients went to her clinic and underwent surgery. FR: Followed up of patients. MMR and FR: Wrote this article.
